# CAR T-cells that target acute B-lineage leukemia irrespective of CD19 expression

**DOI:** 10.1038/s41375-020-0792-2

**Published:** 2020-03-24

**Authors:** Kristen Fousek, Junji Watanabe, Sujith K. Joseph, Ann George, Xingyue An, Tiara T. Byrd, Jessica S. Morris, Annie Luong, Melisa A. Martínez-Paniagua, Khaled Sanber, Shoba A. Navai, Ahmed Z. Gad, Vita S. Salsman, Pretty R. Mathew, Hye Na Kim, Dimitrios L. Wagner, Lorenzo Brunetti, Albert Jang, Matthew L. Baker, Navin Varadarajan, Meenakshi Hegde, Yong-Mi Kim, Nora Heisterkamp, Hisham Abdel-Azim, Nabil Ahmed

**Affiliations:** 1 Interdepartmental Program in Translational Biology and Molecular Medicine, Baylor College of Medicine, Houston, TX USA; 2 Center for Cell and Gene Therapy, Texas Children’s Hospital, Houston Methodist Hospital, Baylor College of Medicine, Houston, TX USA; 3 Texas Children’s Cancer and Hematology Centers, Texas Children’s Hospital, Baylor College of Medicine, Houston, TX USA; 4 Department of Pediatrics, Baylor College of Medicine, Houston, TX USA; 5 Division of Hematology, Oncology and Bone Marrow Transplantation, Children’s Hospital Los Angeles, Los Angeles, CA United States; 6 Department of Chemical and Biomolecular Engineering, University of Houston, Houston, TX USA; 7 University of Southern California Keck School of Medicine, Los Angeles, CA USA; 8 Institute of Medical Immunology, Campus Virchow Klinikum, Charité—Universitätsmedizin Berlin, Berlin, Germany; 9 Berlin Institute of Health—Center for Regenerative Therapies (B-CRT), Charité—Universitätsmedizin Berlin, Berlin, Germany; 10 National Center for Macromolecular Imaging and Verna and Marrs McLean Department of Biochemistry and Molecular Biology, Baylor College of Medicine, Houston, TX USA; 11 Department of Systems Biology, Beckman Research Institute City of Hope, Duarte, CA United States

**Keywords:** Immunotherapy, Tumour immunology

## Abstract

Chimeric antigen receptor (CAR) T-cells targeting CD19 demonstrate remarkable efficacy in treating B-lineage acute lymphoblastic leukemia (BL-ALL), yet up to 39% of treated patients relapse with CD19(−) disease. We report that CD19(−) escape is associated with downregulation, but preservation, of targetable expression of CD20 and CD22. Accordingly, we reasoned that broadening the spectrum of CD19CAR T-cells to include both CD20 and CD22 would enable them to target CD19(−) escape BL-ALL while preserving their upfront efficacy. We created a CD19/20/22-targeting CAR T-cell by coexpressing individual CAR molecules on a single T-cell using one tricistronic transgene. CD19/20/22CAR T-cells killed CD19(−) blasts from patients who relapsed after CD19CAR T-cell therapy and CRISPR/Cas9 CD19 knockout primary BL-ALL both in vitro and in an animal model, while CD19CAR T-cells were ineffective. At the subcellular level, CD19/20/22CAR T-cells formed dense immune synapses with target cells that mediated effective cytolytic complex formation, were efficient serial killers in single-cell tracking studies, and were as efficacious as CD19CAR T-cells against primary CD19(+) disease. In conclusion, independent of CD19 expression, CD19/20/22CAR T-cells could be used as salvage or front-line CAR therapy for patients with recalcitrant disease.

## Introduction

The treatment of B-precursor lymphoid malignancies with chimeric antigen receptor (CAR) T-cells targeting the pan-B cell marker CD19 remains the most impactful application of CAR therapy [1–5]. A substantial number of patients who have failed standard-of-care and salvage therapies, including hematopoietic stem cell transplantation, have achieved durable complete remission using this approach [1–5]. However, as more patients receive CD19CAR T-cells or CD19/CD3 bi-specific T-cell engagers and as long-term follow-up data become available, a high incidence of relapse with CD19(−) disease has been observed [2, 6–8]. It is estimated that this immune evasion mechanism occurs in up to 39% of patients after CD19-directed therapy [3, 7, 9–11].

In order to overcome this limitation, we revisited the exclusivity of CD19 as the sole optimal target antigen for B-lineage acute lymphoblastic leukemia (BL-ALL). We reasoned that other B-lineage markers, such as CD20 or CD22, should be considered as candidate targets. Whereas CD19 is generally ubiquitously expressed, CD20 is expressed in ~50% of cases, and CD22 is expressed in 80–90% [12]. Combinatorial CAR T-cell therapies have shown a clear advantage in preclinical studies of both solid and liquid tumors [13–15]. For example, the bivalent targeting of CD19/CD20 or CD19/CD22 in lymphoma and BL-ALL mitigated antigen escape in murine models [16–19]. Interestingly, we and others have observed that both monovalent and bivalent CD19-specific therapies result in off-target on-tumor antigen modulation, namely partial downregulation of CD20 and CD22 [20]. In addition, a recent clinical trial reported that while 73% of patients with relapsed BL-ALL achieve complete remission after higher dose levels of CD22CAR T-cells, those who relapse do so with CD22(−) or -dim disease [16]. Moreover, paralleling this observation, patients treated with the anti-CD22 antibody drug conjugate inotuzumab ozogamicin have relapsed with CD22-negative disease [21].

In two recent single-patient reports, “sequential loss” of tumor antigen expression has been observed. In diffuse large B-cell lymphoma, one case demonstrated differential loss of CD20, CD30, and CD19 [22] while another showed loss of CD19 and CD22 [23] following immune-based therapies [22, 23]. Furthermore, whereas CD20 expression is often low in BL-ALL, it is expressed by 40–50% of patients with more mature B-lymphoid neoplasms and is associated with poorer prognosis [24, 25]. Specifically, CD20 expression at diagnosis predicts relapse in adult patients, such that CD20-directed antibody therapy is now being incorporated into front-line therapy for adult BL-ALL [25, 26].

Based on these observations, we hypothesized that simultaneous targeting of CD20 and CD22 could confer a therapeutic advantage either as salvage or as upfront CAR therapy [16, 19, 27]. Accordingly, we created a CD19/20/22-targeting CAR T-cell, and we report on its antitumor activity against primary relapsed CD19(+) and CD19(−) escape BL-ALL.

## Materials and methods

### Leukemia samples, blood donors, and cell lines

Primary human samples collection was approved by Institutional Review Boards (IRBs) in accordance with the Declaration of Helsinki. Informed consent was obtained as required by IRBs regulations. UPN01-3 are patient-derived BL-ALL cells, passaged through NOD/SCIDγc−/− (NSG) mice (approved by Institutional Animal Care and Use Committees) and cultured on OP9 cells, as described [28]. Other lines were purchased from American Type Culture Collection (ATCC, Manassas, VA). T-cells were maintained in T-cell media with IL-7/IL-15 as described [29]. BL-ALL were cultured in Alpha MEM, Raji in RPMI, and Daoy and HEK293T in DMEM all supplemented with 1% Glutamax and 10–20% FBS.

### Computational modeling

Individual CAR and antigen models were first generated from amino acid sequences via Swiss-Model Webserver. Initial dockings were done by Patchdock/Firedock (CD19 and CD22CAR/antigen pairs) or based on GA101/CD20 structure (CD20CAR/antigen pair), then refined with Rosetta Dock as described [30].

### Construction of DNA transgenes

Sequences encoding CD19, CD20, and CD22 were obtained from the Research Collaboratory of Structural Bioinformatics Protein Data Base. The CD19-specific single-chain variable fragment (scFv) FMC63 [31] and CD22-specific scFv m971 [32] were previously described. The CD20-scFv was derived from C2B8 and retained part of the constant region, C_L_ and C_H1_ [33]. CAR exodomain sequences were assembled in-frame with a CD8α-hinge and transmembrane domain and 4–1BB and CD3ζ endodomains in Clone-Manager (Sci-Ed, Denver, CO). The CARs were separated by retroviral 2A sequences. DNA sequences were codon-optimized, synthesized by GeneArt (ThermoFisher, Regensburg, Germany), cloned into SFG [34], and verified by pyrosequencing (Epoch, Missouri City, TX).

### Retroviral transduction of cells

T-cells, Daoy, Raji, or UPN03 were transduced with CAR, target, or eGFP.FFLuciferase-encoding transgenes as described [29, 35, 36].

### Flow cytometry and sorting

Accuri-C6, FACSCanto-II, Aria (BD, San Jose, CA), or Gallios (Beckman-Coulter [BC], Brea, CA), were used. Surface staining was performed as described [29]. Data were analyzed using FlowJo software (FlowJo, Ashland, OR). CAR expression was evaluated with CD19CAR FMC63-antibody as described [37]. We used anti-Rituximab (Bio-Rad, Hercules, CA) to detect the CD20CAR, and Fc-conjugated recombinant huCD22 protein (R&D, Minneapolis, MN) (primary) followed by goat anti-human-Fc (Thermo-Fisher, Waltham, MA) (secondary) to assess the surface expression of the CD22CAR. Proliferation was assessed using eFluor670 (eBiosciences, San Diego, CA). Antigens were assessed with CD19, CD20, and CD22 antibodies (BD).

### CRISPR-edited knock-out

CD19 was disrupted in UPN02 and Raji cells by the CRISPR-Cas9 endonuclease system, as described [38]. The primers to generate CD19-specific single-guide RNA were kindly provided by Dr. Lorenzo Brunetti. Three huCD19-specific sgRNAs were produced and electroporation of primary BL-ALL was done as described [38].

### Impedance-based tumor cell killing assay (xCELLigence)

The xCELLigence (ACEA, San Diego, CA) long-term tumor cell killing was used over 100–160 h. Tumor cells were expanded for 18–24 h before T-cells were added. The cell index was monitored every 15-min. A decreasing cell index indicated tumor lysis.

### Cytotoxicity assay

^51^Cr-release assays were previously described [39]. The average lysis of triplicate wells = (test release—spontaneous release)/(max release—spontaneous release) ×100. CAR T-cells were normalized for percentage of transduction.

### Intracellular cytokine staining

BL-ALL and CAR T-cells were cocultured in the presence of Brefeldin A (eBioscience) at 37 °C for 4 h. Cells were fixed (BD Cytofix), permeabilized (BD Perm II), and immunostained for CD45, CD3, CD4, CD8, CD19, IFNγ, and TNFα (BD).

### Imaging flow cytometry

BL-ALL and CAR T-cells were cocultured at 37 °C for 1 h, fixed, permeabilized, then immunostained with CD3, phalloidin, and 7-AAD (Thermo). 1 × 10^5^ events were collected, and samples were analyzed using ImageStream MKII (Luminex, Austin, TX). Acquisition and data analysis were performed using ISX and IDEAS, respectively (Luminex).

### Time-lapse imaging microscopy in nanowell grids (TIMING)

The nanowell manufacture and testing were described previously [40–42]. CAR T-cells and targets were labeled with fluorescent dyes, loaded onto arrays, incubated in media + Annexin V (Invitrogen, Carlsbad, CA), and monitored using a Carl Zeiss Axio Observer (Dublin, CA) fitted with a Hamamatsu (Bridgewater, NJ) Orca-Flash sCMOS camera using a 20 × 0.8 NA objective for 6 h at 5-min intervals. Images were collected and processed for ≥500 wells using an in-house algorithm for cell tracking and segmentation [43].

### CAR-T-cell polyfunctionality evaluation in response to BL-ALL associated antigens by single-cell cytokine profiling

Viable CD8+T-cell subsets were isolated from CAR T-cell products with anti-CD8 microbeads (Miltenyi) and cocultured with Raji cells, Raji-CD19-KO, K562 cells transduced to express CD19 (K562-CD19), or Daoy cells transduced to express either single or combinations of CD19, CD20, and CD22 antigens. The nontransduced CD8+T-cells subjected to the same stimulation were used as a negative control. Subsequent processing, culture conditions, 32-plex antibody barcoded chip analysis, and polyfunctional profiling with determination of polyfunctionality strength index (PSI) and polyfunctional activated topology principal component analysis (PAT PCA) were performed as described [44, 45].

### Animal studies and bioluminescence imaging (BLI)

NSG mice were purchased from The Jackson Laboratory (Bar Harbor, Maine). Sample size not estimated a priori, and experimental arms were unblinded. Mice were ranked based on leukemia engraftment by BLI into high and intermediate, then mice were evenly randomized among all tested groups before T-cell injections. *Raji.CD19KO.eGFP.FFLuc Xenograft:* Raji cells were modified to lose CD19 expression and express eGFP. Firefly luciferase as described above. Cells were sorted on CD19(−)GFP(+) and expanded; 2.5 × 10^5^ tumor cells were tail-vein administered. Engrafted animals were randomly assigned a T-cell group (*n* = 6 each). On day 3, 10 × 10^6^ T-cells were tail-vein injected and tumor burden was tracked by BLI [35]. *rUPN21-R Patient-Derived-Xenograft:* 2 × 10^5^ rUPN21-R primary relapse BL-ALL cells were injected into mice. Animals were randomized into Non-transduced (NT) (*n* = 6), CD19CAR (*n* = 10), and CD19/20/22CAR (*n* = 10) groups; on days 3 and 7 each mouse received 3 × 10^6^ T-cells. Mice were monitored for overall health, and weight was assessed twice/week. Signs of overall decline in health, hind-limb paralysis, and 20% weight loss were used as criteria for euthanasia. *UPN03 Xenograft:* UPN03 primary cells (5 × 10^5^) expressing eGFP. Firefly luciferase were administered intravenously, and all engrafted mice were randomized into treatment groups (*n* = 4–5 per group). On day 9, 5 × 10^6^ T-cells were injected via the tail-vein, and the tumor was quantified over time by BLI.

### Statistical analysis

BL-ALL data were analyzed in Prism v7 software (GraphPad, La Jolla, CA). Data are presented as mean ± standard deviation unless annotated otherwise in figure legends. Variances observed were similar across experimental groups in the results reported. *P* value of <0.05 was considered significant, and the threshold of significance is denoted by *p < 0.05, **p < 0.01, ***p < 0.001, ****p < 0.0001.

## Results

### CD20 and CD22 are heterogeneously expressed in primary BL-ALL and are downregulated in CD19(−) escape

**Fig. 1:**
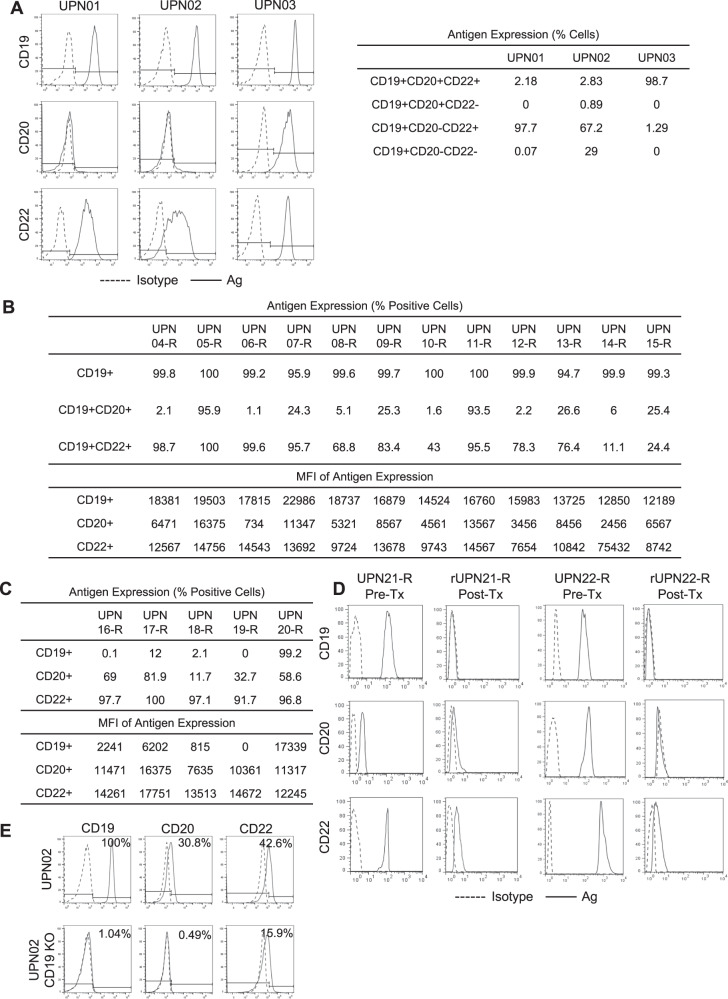
B-lineage ALL expresses variable yet targetable levels of alternative antigens, CD20 and CD22. Flow cytometry was done on ≥30,000 B-lineage BL-ALL cells to assess the expression of CD19 (FITC), CD20 (PE), and CD22 (APC) in each BL-ALL sample. Histograms displayed are representative data. (*n* = 3). **a** Samples from three patients (UPN01, UPN02, and UPN03) were cultured in vitro and assessed for their antigen expression profile. Dotted histogram, isotype; solid line, target antibody. Table displays quantification of antigen expression. **b** Antigen expression in BL-ALL blasts from 12 patients (UPN04-R to UPN15-R) who relapsed after chemotherapy. Quantification of the percentage of antigen-positive cells and the density using mean fluorescence intensity (MFI) are shown in the table. **c** Quantification of CD19, CD20, and CD22 on BL-ALL samples from five patients who relapsed after CD19-directed immune therapy (UPN16-R to UPN20-R). (**d**) B-lineage BL-ALL phenotype before (UPN21-R and UPN22-R) and after CD19CAR T-cell therapy (rUPN21-R and rUPN22-R). (**e**) CD19, CD20, and CD22 expression in UPN02 BL-ALL cells, after CD19 was knocked out using CRISPR/Cas9 technology.

CD19 is near-uniformly expressed on BL-ALL [12]. We examined the antigen expression of three primary BL-ALL cell lines: two Philadelphia (Ph) negative, UPN01 and UPN02, and one Ph + UPN03 (Fig. 1a). All three lines uniformly expressed CD19 but expressed variable levels of CD20 and CD22. In addition, we observed remarkable heterogeneity within each sample with single-, double-, and triple- antigen-positive populations. Similarly, in contrast to uniform CD19 expression, we observed heterogeneity in both percentage and density of CD20 and CD22 in a cohort of 12 patients with BL-ALL who relapsed after chemotherapy (Fig. 1b). Subsequently, we studied samples from five patients who relapsed after CD19-directed immunotherapy and observed loss of CD19 in 4/5, with two (UPN16-R and UPN19-R) exhibiting complete absence of this target antigen while retaining CD20 and CD22 expression (Fig. 1c). In two additional patient samples, we evaluated antigen expression before CD19CAR T-cell therapy (UPN21-R and UPN22-R) and after the emergence of relapse with CD19(−) escape (rUPN21-R and rUPN22-R; Fig. 1d). UPN21-R and UPN22-R expressed high levels of CD19, CD20, and CD22 with varying densities. Upon relapse, rUPN21-R and rUPN22-R lost CD19 expression but preserved CD22 albeit at considerably lower levels. To confirm this observation experimentally, we knocked out CD19 using CRISPR/Cas9 in UPN02 BL-ALL (UPN02 CD19-KO) and confirmed the absence of CD19 from the cell surface using flow cytometry (Fig. 1e). Interestingly, we similarly saw concomitant down-regulation but not complete loss of CD20 and CD22. Based on these findings, we reasoned that CD19(−) recalcitrant disease could be controlled by extending the specificity of therapeutic T-cells to target both CD20 and CD22 simultaneously.

### Generation of a CAR T-cell to cotarget CD19, CD20, and CD22

**Fig. 2:**
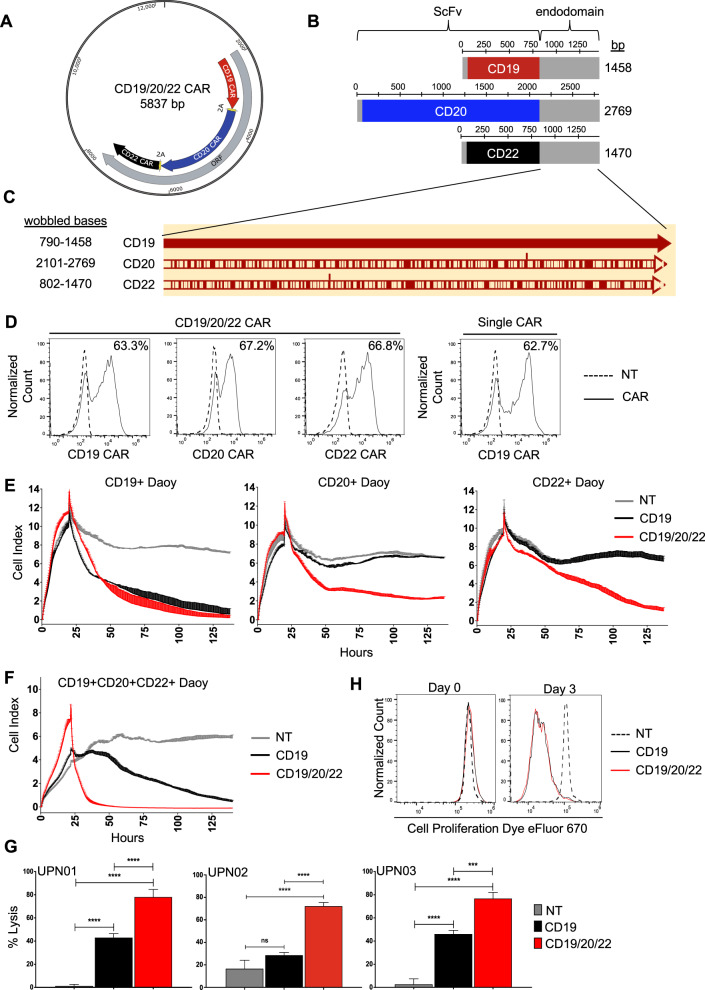
Design of CD19/20/22CAR T-cells. **a** A tricistronic vector was designed with self-cleaving 2A peptides, enabling trivalent protein expression of CD19/20/22-directed CARs on T-cells. **b** Design of CAR transgenes. Each CAR endo-domain contains a CD8α hinge and transmembrane region followed by downstream 4-1BB and CD3ζ intracellular signaling domains. **c** Diagram of DNA wobbling of CAR endo-domain transgenes. Common segments of DNA were wobbled so that no more than 20 consecutive base pairs are the same in any of the three transgenes. Using the CD19CAR sequence as a reference, red bars on the CD20 and CD22 CARs indicate the positions of DNA wobbling. **d** Flow cytometry was performed on T-cells ~1 week after retroviral CAR transduction. Results demonstrate specific binding to each individual scFv region with detection methods unique to each CAR (Fig. S1B). Histograms shown are representative data. Long-term impedance-based xCELLigence killing assay targeting Daoy tumor cells (Fig. S2A, B) expressing each target antigen singly (*n* = 2) (**e**) or all three antigens simultaneously (*n* = 2) (**f**). Tumor cells adhered and expanded for 24 h before CAR T-cells were added in a 1:3 E:T ratio. NT T-cells serve as a negative control. A decreasing cell index indicates tumor lysis. **g** Four hours ^51^Cr release assay targeting B-lineage BL-ALL cells, UPN01, UPN02, and UPN03, at an E:T ratio of 3:1 (*n* = 3). NT T-cells serve as a negative control. Data represent the mean of triplicate samples +SD; *p < 0.05, ***p < 0.001, ****p < 0.0001, one-way ANOVA with Tukey’s multiple comparison post-test. **h** NT, CD19CAR, and CD19/20/22CAR T-cells were stained with eFluor 670 proliferation dye, and proliferation capacity of CAR-expressing T-cells was assessed over 72 h of exposure to BL-ALL cells (*n* = 2).

We engineered a T-cell capable of targeting CD19, CD20, and CD22, using three individual CAR molecules specific for these proteins. As prior literature has demonstrated that the choice of scFv influences CAR functionality [32], we individually modeled the docking of each scFv with its target antigen in silico. CD19/FMC63, CD20/C2B8, and CD22/m971 exhibited favorable electrostatic interaction energies, with minimal adverse cross-reactivity (Table S1; Fig. S1A). To achieve proportionate expression of each CAR on T-cells, we created a single tricistronic transgene encoding the three CAR molecules joined in tandem by 2A-sequences (Fig. 2a). Each CAR exodomain was fused to a hinge and CD8α transmembrane domain followed by a 4–1BB and the T-cell receptor ζ-chain 2nd generation signaling endo-domain (Fig. 2b). To avoid homologous recombination of recurring DNA sequences and secondary RNA structure formation which can lead to poor gene expression [46], we wobbled all similar sequences ≥20 bases (Fig. 2c). The transgene was delivered to donor T-cells using a MoMuLv-based retroviral system, and the surface expression of each CAR exodomain was confirmed by flow cytometry utilizing three distinct scFv-specific methods (Figs. 2d; S1B, C). NT and CD19CAR T-cells served as controls. The CAR T-cell products consisted of a majority (78–86%) of CD8 (+) T-cells (Fig. S1D). To test the ability of CD19/20/22CAR T-cells to distinctly target CD19, CD20, and CD22, we force-expressed these molecules individually on Daoy medulloblastoma cells, which are null for all three antigens (Fig. S2A). Both Daoy and its derivative lines expressed similar levels of immune stimulatory and inhibitory molecules after interferon-γ conditioning (to simulate T-cell activation) and exhibited similar growth dynamics and doubling times (Fig. S3). A long-term killing assay (xCELLIGENCE) demonstrated that while CD19CAR T-cells could kill CD19(+)Daoy but not CD20(+)Daoy or CD22(+)Daoy, CD19/20/22CAR T-cells killed all three lines, confirming their distinct trivalency (Fig. 2e).

We then force-expressed CD19, CD20, and CD22 together on Daoy cells and tested the efficacy of CD19/20/22CAR T-cells, CD19CAR T-cells, and NT in a long-term killing assay. CD19/20/22CAR T-cells eliminated target cells more promptly and sustained this effect over 5 days of testing (Fig. 2f). Next, we tested the cytolytic activity of CD19/20/22CAR, CD19CAR, and NT T-cells against patient-derived BL-ALL: UPN01, UPN02, and UPN03, which have variable expression of the target antigens (see Fig. 1a). CD19/20/22CAR T-cells killed BL-ALL targets better than CD19CAR and NT T-cells (Fig. 2g), yet this enhanced killing capacity was not associated with a more proliferative T-cell phenotype (Fig. 2h).

### CD19/20/22CAR T-cells form a highly functional immune synapse and are more avid serial killers

CD19/20/22CAR T-cells exhibited superior in vitro cytolysis, though not due to a higher proliferative capacity. We therefore investigated whether this advantage could be attributed to more favorable tumor engagement dynamics at the individual T-cell level.

**Fig. 3:**
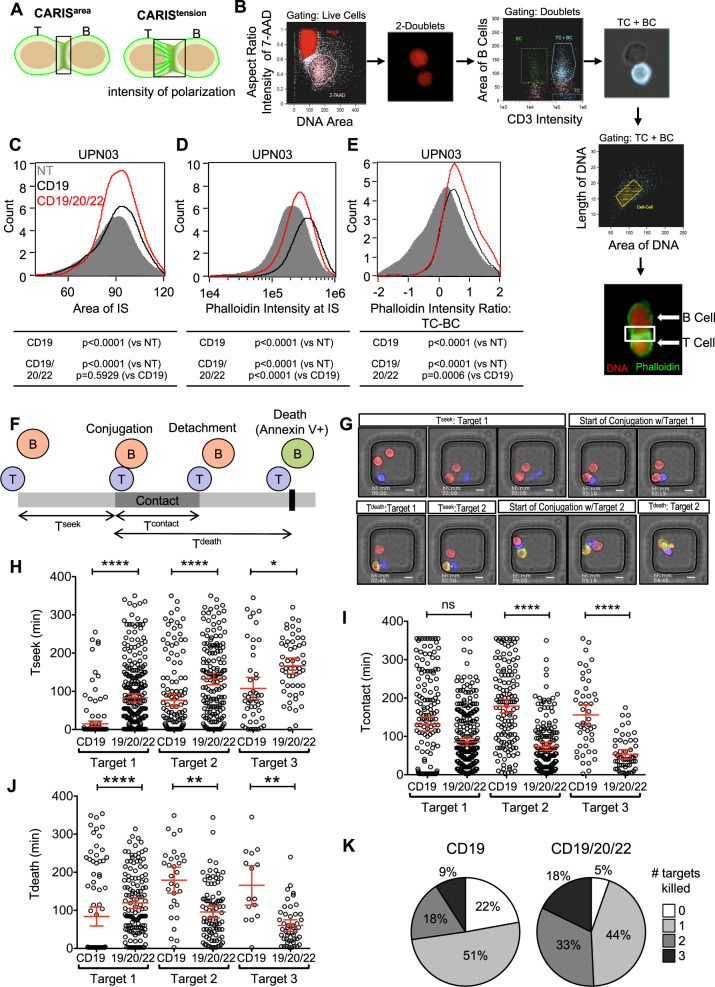
CD19/20/22CAR T cells have increased immunoactivity and serial killing activity at the single-cell level. **a** Schematic illustrating the parameters assessed in the chimeric antigen receptor immune synapse (CARIS). **b** UPN03 cells were cocultured with NT, CD19CAR, or CD19/20/22CAR T-cells at a ratio of 1:1 for 1 h. After incubation, cells were analyzed for expression of CD3 (BV450), phalloidin (FITC), and 7-AAD using ImageStream. (*n* = 3) Duplex cells were separated from single cells by DNA contents and aspect ratio of 7-AAD. Intact T and B (BL-ALL) cells were identified by CD3 intensity and area of CD3(−), respectively. Duplex cells containing both T and B cells were selected for further analysis. Formation of immune synapses between T and B cells were defined by length and area of two DNA clusters between duplex cells. The final image exemplifies the characterization of a CARIS. The DNA is shown as red and F-actin (phalloidin staining) as green. The area boxed in white is the immune synapse (IS) area that is quantified in the analysis. Quantification of (**c**) the area encompassed by the IS (CARIS^area^), (**d**) the intensity of F-actin (phalloidin) at the CARIS (CARIS^density^), and (**e**) the ratio of intensity of phalloidin between T-cells to B-cells (BL-ALL) (CARIS^tension^). The frequency of each parameter for NT (gray), CD19CAR (black), and CD19/20/22CAR (red) T-cells is shown in a histogram. 1 × 10^5^ events were assessed; two-way ANOVA was performed for statistical analysis, and *p* < 0.05 was considered significant. (**f**–**k**) CD19CAR or CD19/20/22CAR T-cells were incubated with UPN02 cells at an E:T ratio of 1:3 to assess their cytolysis activity at the single-cell level using a TIMING nanowell assay. (**f**) Schematic depicting measurements quantified by TIMING assay. (**g**) Microscopy images representing T^seek^, T^contact^, and T^death^ parameters. Scale bars represent 10 μm. (**h**–**j**): Quantification of the time spent (**h**) searching for target by T-cells (T^seek^), (**i**) the duration of contact maintained between an individual CAR T-cell and their first, second, and third target (T^contact^), and (**j**) the time to apoptosis of individual target cells since the start of conjugation (T^death^) at an E:T ratio of 1:3. Each data point represents a single effector cell. ****p < 0.0001, **p < 0.01, *p < 0.05, ns = > 0.05; Kruskall–Wallis test with Dunn’s multiple correction. Data are presented as mean ± 95% confidence interval. (**k**) Pie chart comparing proportion of CD19CAR and CD19/20/22CAR T-cells able to kill multiple targets when plated at an E:T ratio of 1:3. > 165 individual wells were analyzed for each effector cell type.

First, we examined the T-cell/tumor cell interface using high-throughput imaging flow cytometry (ImageStream) to study indices of the CAR Immunological Synapses (CARIS; Fig. 3a) [47]. We probed for actin using phalloidin to quantify the effective CARIS area (CARIS^area^) and intensity (CARIS^intensity^) and the effector/target internuclear distance to assess the tensile properties of the CARIS (CARIS^tension^; Fig. 3a). Our analysis gated on cell doublets of T-cells (CD19/20/22CAR, CD19CAR, or NT) and BL-ALL cells (Fig. 3b). We found that CD19/20/22CAR T-cells engaged BL-ALL cells in CARIS of similar areas (CARIS^area^; Fig. 3c) but of significantly more actin microcluster density (CARIS^density^; Fig. 3d), when compared with CD19CAR or NT T-cells. The distance between the CD19/20/22CAR T-cell nucleus and that of its target were significantly shorter, indicating higher CARIS^tension^ (Fig. 3e). The observed differences in CARIS properties suggest that CD19/20/22CAR T-cells exhibit favorable CARIS cytoskeletal properties, which could enhance their immunoactivity upon engaging BL-ALL cells.

Next, we investigated whether this favorable initial cell interaction leads to superior killing dynamics of individual T-cells. We used high-throughput time-lapse imaging microscopy in nanowell grids (TIMING) assays, which evaluate CAR T-cell engagement and killing dynamics at a single-cell level at various effector to target (E:T) ratios, as previously described [40]. We measured the time T-cells seek BL-ALL cells (T^seek^), the duration in contact (T^contact^), and the time to target death once effector-target conjugation is initiated (T^death^) (Fig. 3f, g and Video S1). CD19/20/22CAR T-cells took significantly longer time to seek target cells and kill their first target compared with CD19CAR T-cells. Thereafter, T^contact^ and T^death^ were significantly shorter with subsequent targets, as CD19/20/22CAR T-cells were able to initiate apoptosis of the BL-ALL cells more efficiently (Fig. 3h–j). Collectively, we observed a higher frequency of second and third (Fig. 3k) serial target killing by a single CD19/20/22CAR T-cell, a higher overall total number of targets killed, and a significantly lower failure rate compared with CD19CAR T-cells. The superiority of CD19/20/22CAR T-cells observed in population-based killing assays could thus be attributable to more efficient anti-leukemic activity of individual CAR T-cells.

### Polyfunctional CD19/20/22CAR T-cells effectively target CD19(−) escape BL-ALL

**Fig. 4:**
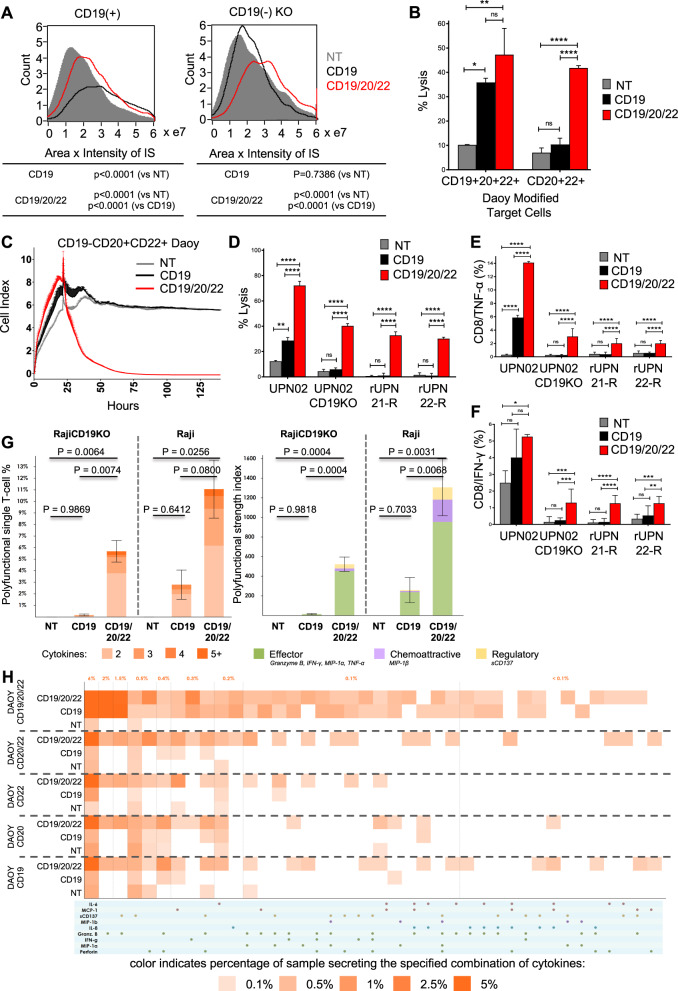
Trivalent CAR T-cells overcome CD19(−) antigen escape in primary BL-ALL. **a** Image stream analysis was performed as described in Fig. 3. The area × intensity of phalloidin in the CARIS is quantified for NT, CD19CAR, and CD19/20/22CAR T-cells in their interaction with CD19-expressing and CD19 KO target cells. **b** Eight hours ^51^Cr release assay targeting cells over-expressing CD19, CD20, and CD22 (triple positive) or CD20 and CD22 (double positive) cells at an E:T ratio of 5:1 (*n* = 2). **c** Long-term impedance-based xCELLigence killing assay targeting double positive (CD20+CD22+) but CD19- cells that are described in Fig. S2B. Target cells were cultured for ~24 h before NT, CD19CAR, or CD19/20/22CAR T-cells were added in a 1:3 E:T ratio. Tumor cell lysis, represented by a decrease in cell index, was measured over time (*n* = 2). **d** Target BL-ALL cells (UPN02, UPN02 CD19KO, rUPN21-R, rUPN22-R) were cocultured with NT or CAR T-cells at an E:T ratio of 3:1 for 4 h and cell lysis was determined by ^51^Cr assay (data shown are representative data, *n* = 3). **p < 0.01, ****p < 0.0001, one-way ANOVA with Tukey’s multiple comparison post-test. BL-ALL target cells (UPN02, UPN02 CD19KO, rUPN21-R, rUPN22-R) were cocultured with T-cells in the presence of brefeldin A at an E:T ratio of 10:1 for 4 h. Cells were stained to determine levels of intracellular (**e**) TNF-α + CD8 + T-cells or (**f**) IFN-γ + CD8+T-cells. NT T-cells serve as a negative control in all experiments (*n* = 3). **p < 0.01, ***p ≤ 0.001, ****p < 0.0001, one-way ANOVA with Tukey’s multiple comparison post-test. (**g**–**h**) CD19+ and CD19- target cells were cocultured with CD8+ isolated CD19/20/22CAR, CD19CAR, and NT T-cells, and single-cell polyfunctionality assessed via a 32-plex antibody barcoded chip analysis. (**g**) Polyfunctionality evaluation of the number and subset classification of cytokines produced by single cells in response to antigen-specific stimulation (**h**) Single-cell functional heatmap demonstrates proportions of polyfunctional subsets of T-cells in response to Daoy cells transduced with various combinations of tumor antigens. Each column corresponds to a specific cytokine or combination of cytokines, and the orange squares represent the frequency at which the cytokine set was secreted by the corresponding sample. The cytokine groups are ordered by overall frequency across all samples.

First, we confirmed that that CD19/20/22CAR T-cells could readily mediate a CARIS formation with both CD19(+) and CD19(−) BL-ALL cells (Fig. 4a). As expected, CD19CAR T-cells failed to mediate such a synapse with CD19(−) BL-ALL cells.

To model the scenario of CD20 and CD22 expression in the absence of CD19, we force-expressed CD20, CD22, and/or CD19 on Daoy and sorted distinct populations based on antigen expression (Fig. S2B). We then tested the cytolytic activity of CD19/20/22CAR, CD19CAR, and NT T-cells against CD19+CD20+CD22+ and CD19-CD20+CD22+targets. CD19/20/22CAR T-cells were significantly better killers of CD19- targets, both in short- (Fig. 4b) and long-term (Fig. 4c) assays compared with CD19CAR T-cells. We then used primary patient leukemia, UPN02, UPN02 CD19-KO, and two post-CD19CAR T-cell escape relapsed BM samples, rUPN21-R and rUPN22-R, as targets and observed that CD19/20/22CAR T-cells induced significantly higher lysis (Fig. 4d) and produced significantly higher levels of the T_H_1 cytokines TNF-α (Fig. 4e) and IFN-γ (Fig. 4f).

Next, we performed single-cell cytokine profiling on the CAR and NT T-cells in the presence and absence of CD19 expression on the target cell, using polyfunctionality in response to BL-ALL associated antigens as a readout. Polyfunctionality is defined as the ability of such T cells to secrete ≥2 cytokines. Individual CD19/20/22CAR T-cells showed greater antigen-specific polyfunctionality upon stimulation with CD19+ (Raji) and CD19− (Raji.CD19KO) targets (Fig. S2C), compared with CD19CAR and NT T-cells (Fig. 4g [left panel] and S4). PAT PCA was used to group polyfunctional cell subsets based on common protein combinations. The common expression of Granzyme B, IFN-γ, MIP-1α, MIP-1β, sCD137 components defined a deeply effector-skewed polyfunctional subset, which drove polyfunctional heterogeneity and was predominately upregulated in CD19/20/22CAR (Fig. S5) compared with CD19CAR and NT T-cells. The PSI, a measure for potency, was significantly higher in CD19/20/22CAR T-cells compared with CD19CAR and NT T-cells (Fig. 4g [Right panel]). The enhanced PSI of the CD19/20/22CAR T-cells was primarily composed of secreted effector proteins. We then tested the single-cell polyfunctional activity of CD19/20/22CAR, CD19CAR, and NT T-cells against single or various combinations of CD19, CD20, and CD22 targets. As shown in Fig. 4h, single-cell functionality profiling showed that CD19/20/22CAR T-cells had increased polyfunctionality compared with CD19CAR or NT T-cells.

These results demonstrate that CD19/20/22CAR T-cells can mediate an effective CARIS with CD19(−) BL-ALL targets and can exhibit robust antitumor immune reactivity and retain considerable polyfunctionality in the face of CD19 loss.

### CD19/20/22 CAR T-cells demonstrate anti-leukemic activity in vivo against CD19(−) and CD19(+) BL-ALL

**Fig. 5:**
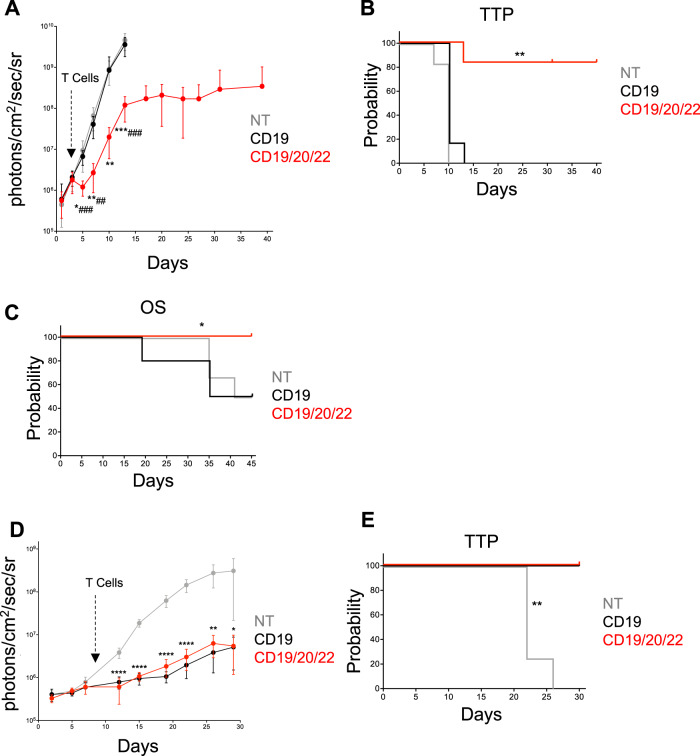
CD19/20/22CAR T-cell efficacy in xenograft models of CD19(−) disease. **a, b** Mice were administered Raji.CD19KO.GFP.FFLuc cells (Fig. S2C) on day 0 followed by NT, CD19CAR, or CD19/20/22CAR T-cells on day 3 (*n* = 6 mice per group). Bioluminescent signal was tracked and quantified over the course of 40 days. **a** The average BLI ± SD for each group is displayed. (*denotes comparisons between NT and CD19/20/22CAR, # denotes CD19CAR vs CD19/20/22CAR; *p < 0.05, **p < 0.01, ***p < 0.001; ## *p* < 0.01, ### *p* ≤ 0.001, one-way ANOVA with Tukey’s post-test). **b** Time to progression (TTP) represented as Kaplan–Meier estimate (tumor burden of 1 × 10^8^ photons/cm^2^/sec/sr considered as disease progression); **p = 0.0013 NT vs CD19/20/22CAR, *p* = 0.0017 CD19 vs CD19/20/22CAR, Gehan-Breslow-Wilcoxon test. **c** Overall survival in mice transplanted with rUPN21-R, a primary BL-ALL exhibiting CD19-escape after CD19CAR T-cell therapy. Injection with rUPN21-R cells on day 0 followed by T-cells on days 3 and 7: NT (*n* = 6 mice), CD19CAR (*n* = 10 mice), or CD19/20/22CAR (*n* = 10 mice). Mice were monitored for signs of disease, weight loss, and general well-being, and overall survival was quantified after 45 days. (*p = 0.0015 NT vs CD19/20/22CAR, *p* = 0.0126 CD19 vs CD19/20/22CAR, Gehan-Breslow-Wilcoxon test). **d,** **e** Mice were administered UPN03.GFP.FFLuc cells on day 0 followed by NT (*n* = 4 mice), CD19CAR (*n* = 5 mice), or CD19/20/22CAR (*n* = 4 mice) T-cells on day 9. **d** Bioluminescent signal (BLI) was recorded and average BLI ± SD quantified over 30 days (*p < 0.05, **p < 0.01, ****p < 0.0001; NT vs CD19 or CD19/20/22CAR; one-way ANOVA with Tukey’s post-test) (E) Time to tumor progression (tumor burden of 1 × 10^8^ photons/cm^2^/sec/sr considered as disease progression) represented as Kaplan–Meier estimate; **p = 0.0047 NT vs CD19CAR and NT vs CD19/20/22CAR, Gehan-Breslow-Wilcoxon Test.

We next evaluated the antitumor efficacy of CD19/20/22CAR T-cells against CD19(−) escape in two models of CD19(−) BL-ALL. First, we modified Raji cells using CRISPR/Cas9 to knockout CD19 and subsequently expressed an eGFP-firefly luciferase (eGFP.FFLuc) reporter gene by retroviral transduction. Cells were sorted based on CD19(−)GFP(+) status (Raji CD19-KO) (Fig. S2C) then engrafted into NSG mice. Mice were treated with CD19/20/22CAR, CD19CAR, or NT T-cells, and bioluminescence imaging (BLI) was used to monitor the tumor burden over time. Only CD19/20/22CAR T-cells mediated an antitumor response, inducing a rapid initial decrease in tumor burden followed by delayed progression (Fig. 5a). All mice treated with CD19CAR and NT T-cells progressed steadily. CD19/20/22CAR T-cells induced a significant delay in tumor progression (Fig. 5b). In addition, we tested CD19/20/22CAR T-cells in mice transplanted with rUPN21-R, a primary B-lineage BL-ALL exhibiting CD19-escape after CD19CAR T-cell therapy. Significant weight loss and hind-limb paralysis were used as signs of progression and as indications for euthanasia. At 45 days, all CD19/20/22CAR mice were alive, whereas 50% of CD19CAR and NT T-cell treated mice had succumbed to disease (Fig. 5c).

Finally, we assess the in vivo efficacy of CD19/20/22CAR T-cells against CD19+ disease compared with CD19CAR T-cells. NSG mice were engrafted with CD19+CD20+CD22+ UPN03 cells transduced with an eGFP-firefly luciferase (eGFP.FFLuc) reporter gene and treated with CD19/20/22CAR, CD19CAR, or NT T-cells. The tumor burden was assessed over time using BLI. The tumors were controlled equally well by CD19/20/22CAR T-cells as by CD19CAR T-cells while NT T-cells failed to exhibit measurable antitumor efficacy (Fig. 5d). CD19/20/22CAR and CD19CAR T-cells also comparably improved the time to progression probability of the treated mice (Fig. 5e). Altogether, these results demonstrate that CD19/20/22CAR T-cells are as effective against CD19-expressing BL-ALL as CD19CAR T-cells but only CD19/20/22CAR T-cells are able to provide tumor control against CD19(−) escape disease.

## Discussion

In general, CD19 is ubiquitously expressed on primary pre-B ALL, and approximately half of cases express CD20, with CD22 high expression on about 80–90% of cases [12]. However, the emerging problem of CD19(−) escape after CD19-directed immunotherapies [2, 3, 6–11] compromises the success of these breakthrough approaches. Similarly, even CD22(−) escape was reported in a phase 1 clinical trial of CD22CAR T-cells [16]. Here, we found that following CD19CAR T-cell therapy, expression of CD19 varied from ubiquitous (e.g., UPN20-R) to, more commonly, less than 10% of cells. On the other hand, CD22 and CD20 were expressed at varying percentages in samples from both diagnosis and relapse, as reported by others [20]. Thus, CD19(−) escape variants downregulate but not altogether eliminate CD20 and CD22 cell surface expression. We conclude that CD19, CD22, or CD20 target densities are unpredictable and likely to vary in relapsed patients. This raises the question if a minimum antigen threshold exists to provoke a productive CAR T-cell response. Although a signal threshold does appear to exist for T-cell receptor-mediated signaling, much less is known regarding T-cells expressing a CAR. One study reported that the combination of CAR density and tumor antigen density regulated T-cell potency in mouse models [48] but overall little is known. Hamieh et al. [49]. more recently addressed this important point in depth by comparing CAR T-cell activities against NALM6 cells with mono- or biallelic knockout of CD19, finding that the configuration of the intracellular CAR domain [-BBζ vs −28ζ] together with the target density of CD19 determined the threshold efficacy for CAR T-cell activation and target cell lysis. Interestingly, the authors also showed that a combination of CD19CAR-28ζ with CD22CAR-BBζ CARs expressed in the same T cell provided a significant benefit in mouse models with low Nalm6 CD19 and CD22 target densities. These results concur with our studies and suggest that the addition of CD20 in our product is likely to further mitigate the deleterious effects of low target densities on the target cell. Taking these findings into account we used a variety of primary BL-ALL samples and models with differential CD19, CD20, and CD22 expression profiles as observed in patients and generated a trivalent CAR T-cell targeting all three antigens.

Our CAR T-cell design incorporated a number of features to support high CAR expression. First, we used in silico modeling that relies on analysis of the stereo-electric coaptation of the three CAR recognition exodomains to avoid detrimental conformations in this complex biologic as used here. One potential confounding factor was that we needed to include three CAR endodomains in a single DNA construct. As previously described, homologous recombination between the repeated sequences and increased RNA secondary structure formation may be problematic with this approach [46]. Thus, to optimize the production of the three identical CAR endodomains we “wobbled” –namely, used alternative DNA coding sequences that transcribe into the same amino acid sequence [46]. This strategy optimizes protein production and increases the effective surface expression of each distinct CAR. Indeed, the three CARs were expressed at similarly high levels (63–67%) on the surface and were comparable to the expression of the single CD19CAR. Finally, to facilitate clinical translation, we used a single transgene to express the three CAR molecules, which theoretically decreases the risk of insertional mutagenesis compared with repeated, separate integrations of multiple genes and associated promoters into the T-cell genome.

When we compared the CD19/20/22CAR to the CD19 single CAR using in vitro functional testing against target cells, CD19/20/22CAR T-cells exhibited superior cytolytic activity compared with CD19CAR T-cells, both against modified Daoy cells and against three primary BL-ALLs with differential expression of CD20 and CD22. We observed differences in the kinetics of CAR T-cell interactions with BL-ALL cells that could explain the superior killing activity of CD19/20/22CAR T-cells, and indeed they were significantly better serial killers. Specifically, as the CARIS and its important structural component F-actin are critical for target killing [47], we quantitated F-actin in the IS and the intensity of cell polarization. The CD19/20/22CAR CARIS contained more F-actin, and the nuclei of target and T-cells reached closer proximity compared with target and CD19CAR T-cell nuclei. We speculate that this could be a reason why CD19/20/22CAR T-cells needed more time to repolarize, engage and form a CARIS, and also disassemble and reassemble the structures needed to support target cell lysis and switch to the migratory program, resulting in a longer T_seek_ time than CD19CAR T-cells. These combined properties of CD19/20/22CAR T-cells, which form a synapse that is stronger but has a shorter span of existence, suggest that some T-cells can integrate the strength of the CARIS with the contact time to produce a uniform cytotoxic response. The latter could explain the performance of individual CAR T-cells upon polyfunctional profiling. When evaluated on the single-cell level, CD19/20/22CAR T-cells had significantly higher polyfunctionality, as measured by PSI, against CD19+ and CD19− targets than CD19CAR T-cells. In previous studies, higher PSI values were associated with higher effector cell potency in in vivo preclinical models [50], revealed differences in CAR T-cell product optimization [51], and predicted patient response to CAR T-cell therapy [44]. Together, the CARIS and PSI findings support a potential advantage of CD19/20/22CAR in clinical applications.

On a functional level, the assessment of T-cell activation upon target encounter collectively showed that CD19/20/22CAR T-cells are adaptively more activated upon encounter of their target(s). We evaluated their phenotypic activation/exhaustion profile and observed that CD19/20/22CAR T-cells are more activated upon encounter of either CD19+ or CD19+CD22+CD22+ when compared with CD19CAR T-cells (Fig. S6A, B). The expression levels of PD-1 and LAG-3 on CD19/20/22CAR T-cells upon encounter of CD19+ targets were comparable to CD19CAR T-cells, yet they exhibited more PD-1 and LAG-3 only upon encounter of CD19+CD20+CD22+targets (Fig. S6C, D). Interestingly, we observed precipitous CD19CAR downregulation in both CD19/20/22CAR T-cells and CD19CAR T-cells upon encounter of CD19+targets. Only CD19/20/22CAR T-cells maintained CD20 and CD22 CAR expression and consequently their activity against these target molecules (Fig. S7). The expression of activation/exhaustion molecules has traditionally been used to describe a phenotype of immune cells, yet the functional indices of activation (cytokine, killing, multiplex cytokine) and sustenance of functionality in the face of exhaustion (individual cell serial killing, xCelligence, and animal experiments) should be considered for a more complete functionality profile.

To confirm the advantages observed in our in vitro findings, we tested the activity of the CD19/20/22CAR T-cells in vivo. We found that in NSG xenograft models, the CD19/20/22CAR T-cells were able to overcome the inability of CD19CAR T-cells to control growth of CD19(−) leukemia cells from patients who failed CD19CAR T-cell therapy, as well as of the genetically-modeled CD19(−) escape variants (Raji.CD19KO). Importantly, CD19/20/22CAR T-cells were as efficacious as CD19CAR T-cells against primary relapse CD19(+) BL-ALL. Simultaneous targeting of CD19 and CD22 reduced the risk of CD19- relapse compared with “single targeting” in a human study reported by Wang et al. [52]. In this work studying the efficacy and safety of CAR19/22 T-cell cocktail therapy in patients with refractory/relapsed B-cell malignancies, only one of 89 patients (1.1%) relapsed with CD19 negative disease with a median follow-up of 14.4 months (range, 0.4–27.4). This is in sharp contrast to the reports of CD19 negative relapse (18–25%) from various clinical trials infusing CD19CAR T-cells [53]. To rigorously test whether the CD19/20/22CAR T-cell would reduce the upfront chance of CD19-/lo relapse, a similarly designed human trial would be warranted.

CAR T-cells that target multiple tumor antigens were shown in our previous work to overcome low levels of tumor antigen expression [29]. For the specific treatment of BL-ALL, we have increased the activity and broadened the spectrum of CD19CAR T-cells by targeting two additional antigens and extended the therapeutic reach of the T-cell product to other CD20-expressing lympho-reticular malignancies. Thus, a single CAR T-cell product targeting all three antigens provides the advantage of applicability to a broader population of patients with BL-ALL and to a more comprehensive range of other B-lineage malignancies, which routinely express higher levels of CD20 [54]. We note that although the target density of CD19, CD20, and CD22 antigens on individual diagnosis and relapsed patient cells is unpredictable, and the degree to which CD19, CD20, or CD22 are targeted individually cannot be deduced from our data, our results suggest these may not be key factors relevant for effective cell killing by our trivalent CAR T-cells. Thus, although the toxicity of our CAR T-cell product will need to be assessed in carefully designed human trials, the effectiveness of CD19/20/22CAR T-cells against CD19(−) escape BL-ALL and CD19(+) BL-ALL alike compares very favorably to that of the benchmark CD19CAR T-cells.

## Supplementary information

Supplemental Text

Supplemental Table 1

Supplemental Figure 1

Supplemental Figure 2

Supplemental Figure 3

Supplemental Figure 4

Supplemental Figure 5

Supplemental Figure 6

Supplemental Figure 7

Supplemental TIMING Video
