# Supplementary material for: CAR T-cells that target acute B-lineage leukemia irrespective of CD19 expression
Source: Leukemia. 2020 Mar 24;35(1):75–89. doi: 10.1038/s41375-020-0792-2 (PMC7519582; doi:10.1038/s41375-020-0792-2)
Supplement: Supplementary file 1 — Supplemental Text [file 41375_2020_792_MOESM1_ESM.pdf]

Supplemental Tables:

Table S1. CAR-Antigen Interaction Properties

Description of CAR-Antigen interaction properties, including the total number of protein residues modeled, the amount of residues interacting at the protein interface, quantification of the solvent-accessible area at the interface, and identification of key bonds involved in the binding at the interface.

Supplemental Figure Legend:

Figure S1. CD19/20/22CAR Design and Detection on T-cells

(A) *In silico* protein models illustrating the predicted interactions between each CAR and its respective antigen (CD19CAR - red-orange, CD19 - grey, CD20CAR - blue, CD20 - green, CD22CAR - orange, CD22 - purple). Modeling of the electrostatic interactions between the positively charged (blue) and negatively charged (red) regions of the CAR and antigen within the binding pocket. (B) Cartoon depicting the staining strategies for detecting the scFv region of each individual CAR molecule. (C) Flow cytometry plots illustrating that individual CD19/20/22CAR T-cells either express all three CARs simultaneously or remain as a non-transduced cell. CARs are not expressed at different levels. Live T cells were first gated on CD20CAR positive or negative cells. These two cell populations were then assessed for CD19CAR and CD22CAR expression. (D) NT, CD19CAR, and CD19/20/22CAR T-cells were assessed by flow cytometry for their expression of CD4 and CD8 co-receptors.

Figure S2. Phenotype of Model Cell Lines: Daoy and Raji

(A) Daoy tumor cells (natively CD19-CD20-CD22-) were stably modified to express each of the antigens of interest individually and sorted for CD19+, CD20+, or CD22+ cells, respectively. (B) Daoy cells were modified to be CD19+CD20+CD22+ triple positive or CD20+CD22+ double positive and sorted for these triple- and double-positive populations,

respectively. Flow cytometry dot plots exhibit the cell populations that were collected and used in the following assays. (C) Raji cells were modified to express a GFP.FFLuciferase gene, and CD19 was knocked out by using CRISPR/Cas9 technology. Raji and Raji.CD19KO cells were sorted based on GFP(+) or GFP(+)CD19(-) status, respectively, and their expression of CD19, CD20, and CD22 was assessed by flow cytometry.

Figure S3. Immune ligand profile and growth characteristics of Daoy WT and antigen-modified cell lines. (n=3) (A) Flow cytometry plots exhibiting the level of expression and quantification of the immune activation ligands HLA-DR (MHC II), CD80, and CD86 on Daoy, CD19+ Daoy, and CD19+CD20+CD22+ Daoy cells at baseline and in response to IFN-γ. (B) Flow cytometry plots and quantification of the expression of immune inhibitory ligands PD-L1, PD-L2, and CD40L on Daoy, CD19+ Daoy, and CD19+CD20+CD22+ Daoy cells at baseline and in response to IFN-γ. Quantification was determined using the mean fluorescence intensity for each marker. (C) An impedance based xCELLigence assay was used to measure the growth kinetics of Daoy WT and Daoy lines expressing CD19 alone or CD19, CD20, and CD22. The cell index is graphed as a measure of cell line growth kinetics over the course of 3 days. Quantification of the doubling time of each cell line over the course of 70 hours is provided in the bar graph.

Figure S4. Single-cell functional heatmap of CAR T-cell responses against CD19+ and CD19- targets

Single-cell functional heatmap reveals enhanced polyfunctional cell subsets with distinct protein combinations across CAR T-cell products upon antigen specific stimulation. Each column corresponds to a specific cytokine or combination of cytokines, and the orange squares represent the frequency at which the group was secreted by the corresponding sample. The cytokine groups are ordered by overall frequency across all the samples.

CD19/20/22CAR shows the greatest increase of polyfunctional cell subsets in response to CD19KO or WT Raji cells or K562-CD19 cell stimulation compared to NT or CD19.

Figure S5. Single-cell PAT PCA of CAR T-cell responses against CD19+ and CD19- targets

Single-cell PAT PCA shows complex yet distinct polyfunctional landscaping among the CAR-T products in response to antigen-specific stimulation. Dots represent single-cells, and broader circles are color weighted to dominance of a functional protein/cytokine subset in each sample. Polyfunctional cell subsets with various protein combinations were predominately upregulated in CD19/20/22CAR in response to Raji cell stimulation (olive green) compared to all other stimulated conditions. Granzyme B, IFN-γ, MIP-1α, MIP-1β, and sCD137 components indicate a deeply effector-skewed polyfunctional subset and drive polyfunctional heterogeneity.

Figure S6. Expression of phenotypic T-cell markers of immune stimulation and exhaustion. (n=3) NT, CD19CAR, and CD19/20/22CAR T-cells were co-cultured with Daoy, CD19+ Daoy, and CD19+CD20+CD22+ Daoy target cells at an E:T ratio of 1:1. T-cells were collected at 24 hours and 48 hours for assessment of CD25 and at 96 hours for evaluation of PD-1 and LAG-3. (A) Flow cytometry plots exhibiting the expression of CD25 at 0 (baseline), 24, and 48 hours of stimulation. (B) Quantification of the fold change in the MFI of CD25. (C) Flow cytometry plots demonstrating the level of expression of PD-1 and LAG-3 on NT, CD19CAR, and CD19/20/22CAR T-cells at 0 (baseline) and 96 hours of stimulation. (D) Quantification of the fold change in the MFI of PD-1 and LAG-3.

Figure S7. Surface expression of CARs on CD19/20/22CAR T-cells in response to CD19+ target cells. CD19/20/22CAR T-cells were co-cultured with WT or CD19+ Daoy target cells at an E:T ratio of 1:1. Flow cytometry was performed for surface expression of

each individual CAR at 0 (baseline), 4, and 24 hours. (n=3) Flow histograms display the change in surface expression of each specific CAR on CD19/20/22CAR T-cells.

Supplemental Video:

Video S1. Representative video of nanowell examined in TIMING assay. Representative example of serial killing by a CD19/20/22CAR T-cell (labeled in blue) co-cultured with two target cells (labeled in red) in nanowell grid. The death marker is in green. Scale bar is 10 μm. Time is displayed as hh:mm, and the video is sped up 900X.

Supplementary Methods:

Computational Modeling

In the case of the CD19 and CD22 CAR/antigen pairs, the top 100 dockings in Firedock were screened based on docking quality, as well as agreement with known biochemical and structural data before the top docking pairs were further refined with Rosetta Dock via Rosie. Results from Rosetta Dock were screened based on the total score, rms deviation, and the interface energy scores from Rosetta Dock, as well as the agreement with known structural and biochemical data. In the initial model of the CD20 CAR/antigen pair the CD20 CAR model was structurally aligned to the GA101 chain bound to the CD20 antigen structure before further refinement with Rosetta Dock. Final candidate dockings were examined in UCSF Chimera and analyzed using PISA and PDBSUM from PDBE.

Model	Template (PDB ID)	Sequence identity (%)	Coverage (range)	GMQE	QMean
CD19 CAR	3ESV A	67.08	49% (22-263)	0.38	-0.98
CD20 CAR	1HZH B	88.24	48% (22-463)	0.48	0.97
CD22 CAR	5YAX B	76.99	49% (22-266)	0.42	-1.35
CD19 antigen	5AAW A	19.80	36% (21-266)	0.13	-5.11
CD20 antigen	3PP4 C	100.00	8% (164-187)	0.05	.30

CD22 antigen	3DMK A	18.24	80% (29-677)	0.40	-5.62

Detection of CAR Expression

CAR expression was evaluated with flow cytometry staining. To detect the CD19CAR a PE-conjugated anti-idiotype monoclonal antibody which binds specifically to the FMC63 ScFv was used[1]. For detection of the CD20CAR a FITC-conjugated anti-idiotype anti-Rituximab antibody was used (Bio-Rad, Hercules, CA). Due to the unavailability of the latter reagent, we used a human CD20.Fc (R&D, Minneapolis, MN) followed by an anti-Fc secondary antibody (Thermo-Fisher, Waltham, MA) in the experiment described in Supplemental Figure 7. The CD22CAR was detected by a two-step method using a Fc-conjugated human recombinant CD22 protein (R&D, Minneapolis, MN) in the primary step followed by an APC-conjugated goat anti-human Fc antibody (Thermo-Fisher, Waltham, MA). Each detection method is able to specifically detect the ScFv region of the individual CARs. The cartoon depicting the staining strategy on CD19/20/22CAR T-cells (Figure S1B) was created with BioRender.

CRISPR-mediated knock-out

The CD19-specific sgRNAs were produced *in vitro* as previously described.[2] A total of 9.9 μg in-vitro transcribed single-guide RNA (3.3 μg each) was mixed with 10 μg recombinant Cas9 protein (PNAbio, Newbury Park, CA) and transfected into two million primary B-lineage ALL cells using the Neon Electroporation Kit (100 μL tip, Invitrogen, Carlsbad, CA). The electroporation was executed at 1650V for 10 ms with 3 pulses. After transfection, the electroporated cells were added directly into antibiotic-free medium and rested for 24 hours. After 7 days, the knockout was confirmed using flow cytometry, and CD19(-) cells were sorted using an Aria (BD) sorter.

Guide RNA Sequences

Primer Name	Sequence (5'-3')
CD19ex3#58	ttaatacgactcactataGGGCCCCAAGCTGTATGTGTgttttagagctagaaatagc
CD19ex4#89	ttaatacgactcactataGGGACCCATGTGCACCCCAAgttttagagctagaaatagc
CD19ex4#89b	ttaatacgactcactataGGGTCTTGAGCTGTGGCCCGgttttagagctagaaatagc
Universal Rev	AGCACCGACTCGGTGCCACT

Imaging cytometry analysis (ImageStream)

Immune synapses were identified by selecting: 1) Area of 7-AAD vs Aspect Ratio of 7-AAD (cell-cell complexes), 2) intensity of CD3 vs Area of Mask for CD3(-) cells (T-cell-B-cell complexes), and 3) Area vs Length of mask for duplex DNA content (tight duplex T-cell-B-cell interactions, excluding multiplex complexes). Masks for immune synapses were created using the Valley mask option between DNA contents between 2 cells.

Time-lapse Imaging Microscopy in Nanowell Grids (TIMING) Assay

CAR+ T-cells and target cells were labeled with 2 μM PKH 67 and 2 μM PKH 26 (Sigma Aldrich, St. Louis, MO), respectively. The effectors and targets were loaded sequentially onto nanowell arrays at a concentration of 0.5×10^6 cells/mL and 1×10^6 cells/mL, respectively, and the entire nanowell array was incubated in complete media containing Annexin V-conjugated with Alexa Fluor™ 647 (Invitrogen, Carlsbad, CA) at 37°C/5% CO_2.

CAR T-cell polyfunctionality evaluation in response to BL-ALL associated antigens by single-cell cytokine profiling

Non-transduced, CD19CAR and CD19/20/22CAR T-cells were generated from the same donor. Viable CD8+ T-cell subsets were isolated from CAR T-cell products with anti-CD8

microbeads (Miltenyi)[3,4] and then cocultured with Raji cells, RajiCD19KO, K562 cells transduced to express CD19 (K562-CD19) or Daoy cells transduced to express either CD19, CD20, or CD22 as a single antigen, with combination of CD20/22 or CD19/20/22 antigens, at a 1:2 E:T ratio for 20 hours at 37°C, 5% CO_2. The non-transduced T-cells from the same stimulation were used as a negative control. After stimulation, the co-cultured CD8+ T-cells were enriched by the depletion of target cells by using anti-CD19 or CD20 conjugated magnetic beads. CD8+ CAR T-cells were stained with Alexa Fluor 647 conjugated anti-CD8 antibody at room temperature for 10 minutes, rinsed once with phosphate-buffered saline (PBS), and resuspended in complete RPMI medium at a density of 1 x 10^6/mL. Approximately 30 µL of cell suspension was loaded into the IsoCode Chip and incubated at 37°C, 5% CO_2 for additional 16 hours. Protein secretions from ~1000 single T-cells were captured by the 32-plex antibody barcoded chip and the polyfunctional profile was analyzed by the IsoSpeak software across the five functional groups:

Effector: Granzyme B, TNFα, IFN-γ, MIP1α, Perforin, TNFβ;

Stimulatory: GM-CSF, IL-2, IL-5, IL-7, IL-8, IL-9, IL-12, IL-15, IL-21;

Chemoattractive: CCL11, IP-10, MIP-1β, RANTES;

Regulatory: IL-4, IL-10, IL-13, IL-22, sCD137, sCD40L, TGFβ1;

Inflammatory: IL-6, IL-17A, IL-17F, MCP-1, MCP-4, IL-1β.

The PSI of T-cells was computed using a pre-specified formula, defined as the percentage of polyfunctional cells, multiplied by mean fluorescence intensity (MFI) of the proteins secreted by those cells and polyfunctional activated topology principal component analysis (PAT PCA) was performed as described before.[3,4]

$$\mathrm{PSI_{sample}} = (\%\ \text{polyfunctional cells in sample}) \sum_{i=1}^{32} \text{MFI of secreted protein i of the polyfunctional cells}$$

PSI is defined as the percentage of polyfunctional single-cells (secreting 2 or more proteins) in a sample, multiplied by the average signal intensity of the secreted proteins from individual functional groups from each cell. Each cell's strength, across 1000+ cells, is then aggregated and simplified into the read of PSI. The PSI measurement provides a comprehensive visualization of the potent cell subsets, and the cytokine types driving these potent cell subsets. The averaged data were generated from at least 3 sections of each chip. The statistical analysis among the groups was done by one-way ANOVA.

Flow Cytometry Analysis

Phenotypic markers on T-cells: Tumor cells were cultured overnight in 96-well plates. T-cells were added at a 1:1 ratio after normalizing for CAR expression. T-cells were collected at 24 and 48 hours to detect the activation marker CD25 (FITC) (BD). T-cells were collected at 96 hours to detect activation and exhaustion markers PD-1 (PE-Cy7) (Biolegend) and LAG-3 (BV421) (BD). *Co-stimulatory and inhibitory tumor ligands:* 2.5×10^5 cells were seeded in complete DMEM +/- 10 ng/ml IFN-γ (BioLegend, USA). Cells were harvested 24 hours later and probed for HLA-DR (FITC), CD80 (PE), and CD86 (APC), or PD-L1 (PE-Cy7), PD-L2 (APC), and CD40L (PE) (BD, San Jose, CA). All compensation controls were done using single antibody probed compensation beads, and samples were analyzed using BD Canto II. Flow cytometry data was analyzed using FlowJo software (v.10).

References

1. Jena B, Maiti S, Huls H, et al. Chimeric antigen receptor (CAR)-specific monoclonal antibody to detect CD19-specific T cells in clinical trials. *PLoS One*. 2013;8(3):e57838.
2. Gundry MC, Brunetti L, Lin A, et al. Highly Efficient Genome Editing of Murine and Human Hematopoietic Progenitor Cells by CRISPR/Cas9. *Cell Rep*. 2016;17(5):1453-1461.

3. Rossi J, Paczkowski P, Shen YW, et al. Preinfusion polyfunctional anti-CD19 chimeric antigen receptor T cells are associated with clinical outcomes in NHL. *Blood*. 2018;132(8):804-814.
4. Xue Q, Bettini E, Paczkowski P, et al. Single-cell multiplexed cytokine profiling of CD19 CAR-T cells reveals a diverse landscape of polyfunctional antigen-specific response. *J Immunother Cancer*. 2017;5(1):85.
