# Supplementary material for: CAR T-cells that target acute B-lineage leukemia irrespective of CD19 expression
Source: Leukemia. 2020 Mar 24;35(1):75–89. doi: 10.1038/s41375-020-0792-2 (PMC7519582; doi:10.1038/s41375-020-0792-2)
Supplement: Supplementary file 2 — Supplemental Table 1 [file 41375_2020_792_MOESM2_ESM.pdf]

Supplemental Table 1

CAR-Antigen Interaction Properties		
	**CAR**	**Antigen**
**CD19**		
Total residues	242 (100%)	243 (100%)
Residues at interface	29 (12%)	29 (11.9%)
Solvent-accessible area at interface	839.2 Å (7.1%)	863.3 Å (6%)
Bonding at interface	5 H bonds	
**CD20**		
Total residues	222 (100%)	24 (100%)
Residues at interface	15 (6.8%)	8 (33.3%)
Solvent-accessible area at interface	326.7 Å (2.7%)	367.4 Å (15.6%)
Bonding at interface	3 H bonds	
**CD22**		
Total residues	245 (100%)	649 (100%)
Residues at interface	34 (13.9%)	31 (4.8%)
Solvent-accessible area at interface	949.4 Å (7.1%)	864.2 Å (2.3%)
Bonding at interface	2 H bonds & 2 salt bridges	
