# Supplementary figures and images for: CAR T-cells that target acute B-lineage leukemia irrespective of CD19 expression
Source: Leukemia. 2020 Mar 24;35(1):75–89. doi: 10.1038/s41375-020-0792-2 (PMC7519582; doi:10.1038/s41375-020-0792-2)

Supplemental Figure 1

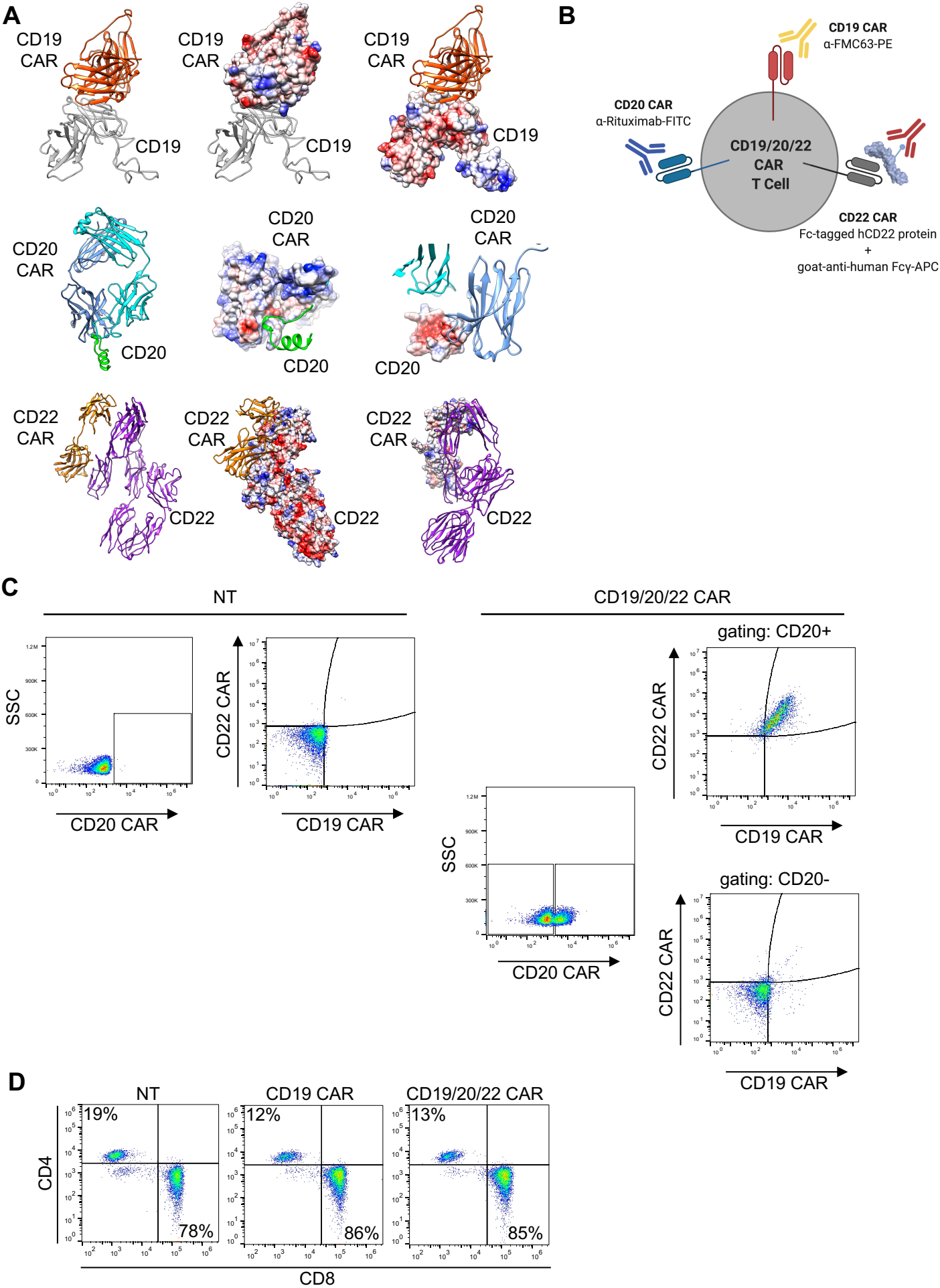

Supplement: Supplementary file 3 — Supplemental Figure 1 [file 41375_2020_792_MOESM3_ESM.pdf]

Supplemental Figure 2

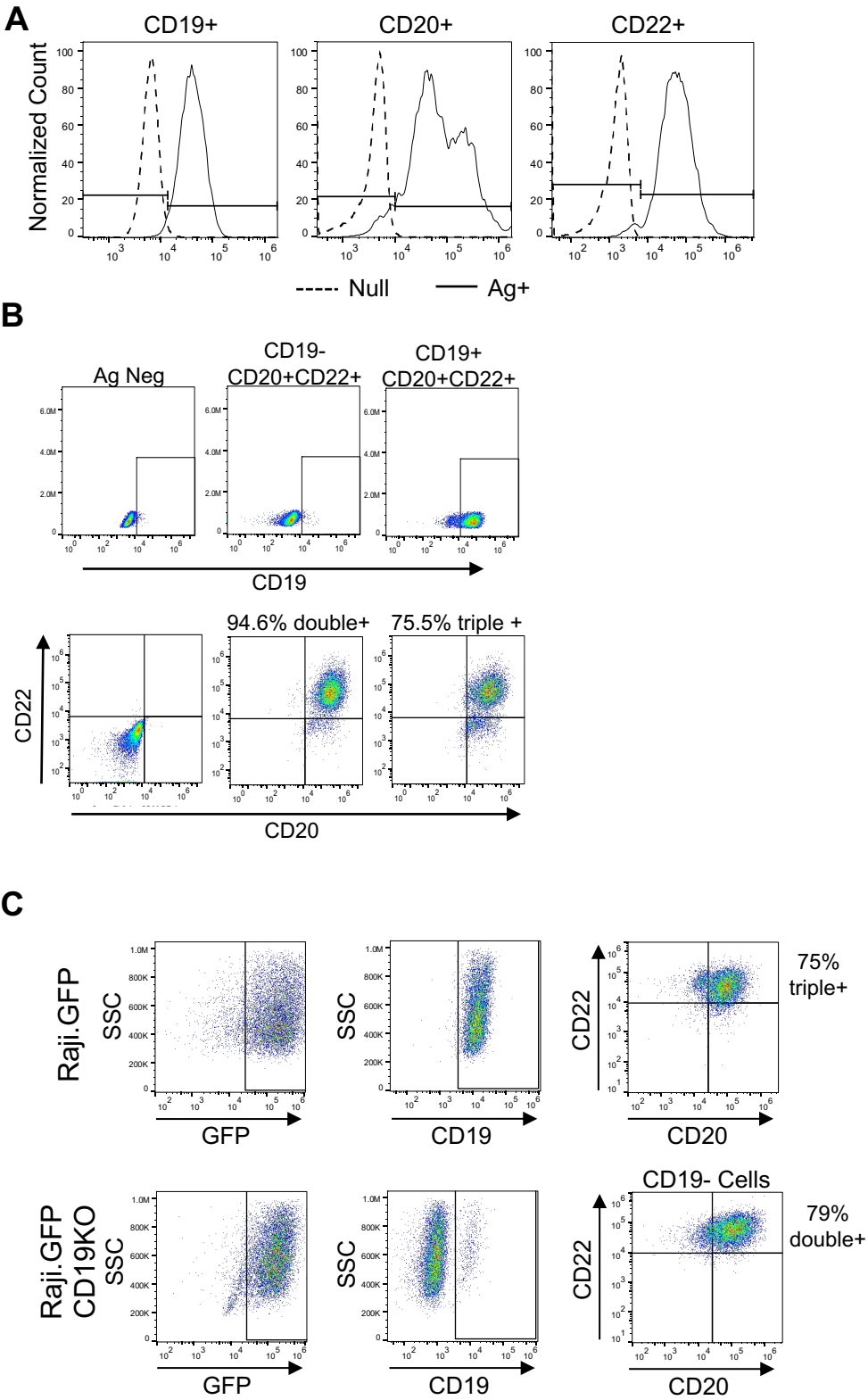

Supplement: Supplementary file 4 — Supplemental Figure 2 [file 41375_2020_792_MOESM4_ESM.pdf]

Supplemental Figure 4

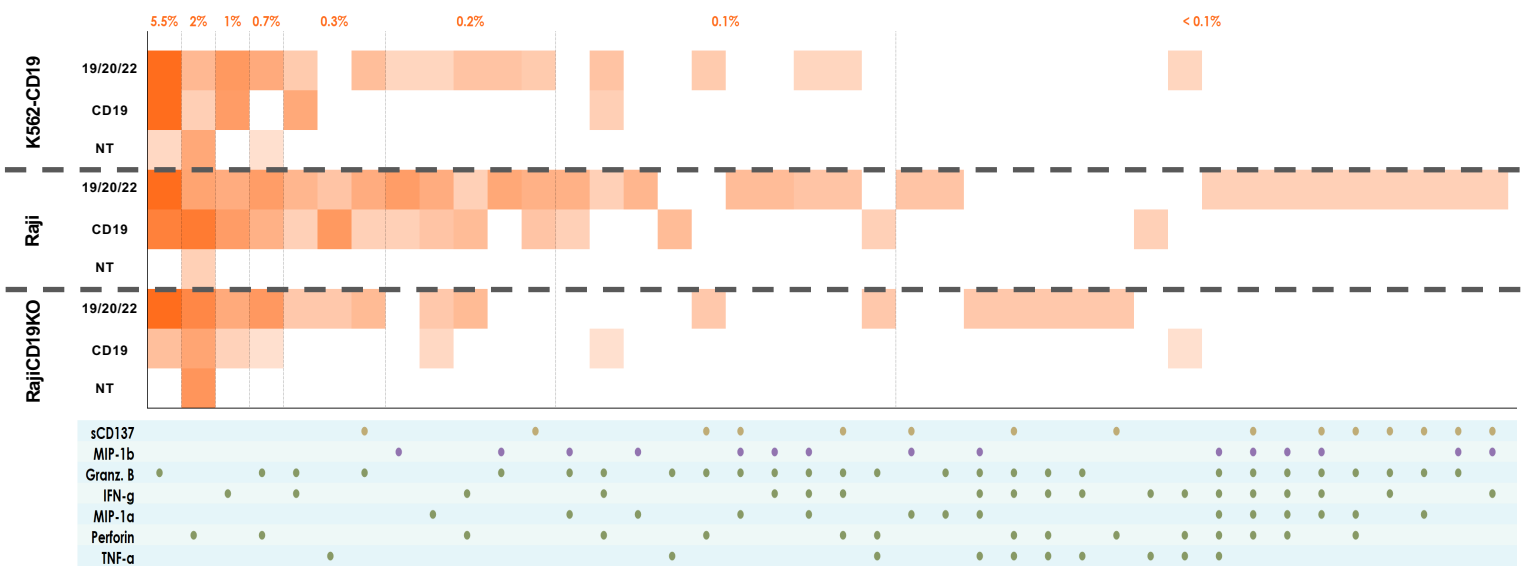

Supplement: Supplementary file 6 — Supplemental Figure 4 [file 41375_2020_792_MOESM6_ESM.pdf]

Supplemental Figure 5

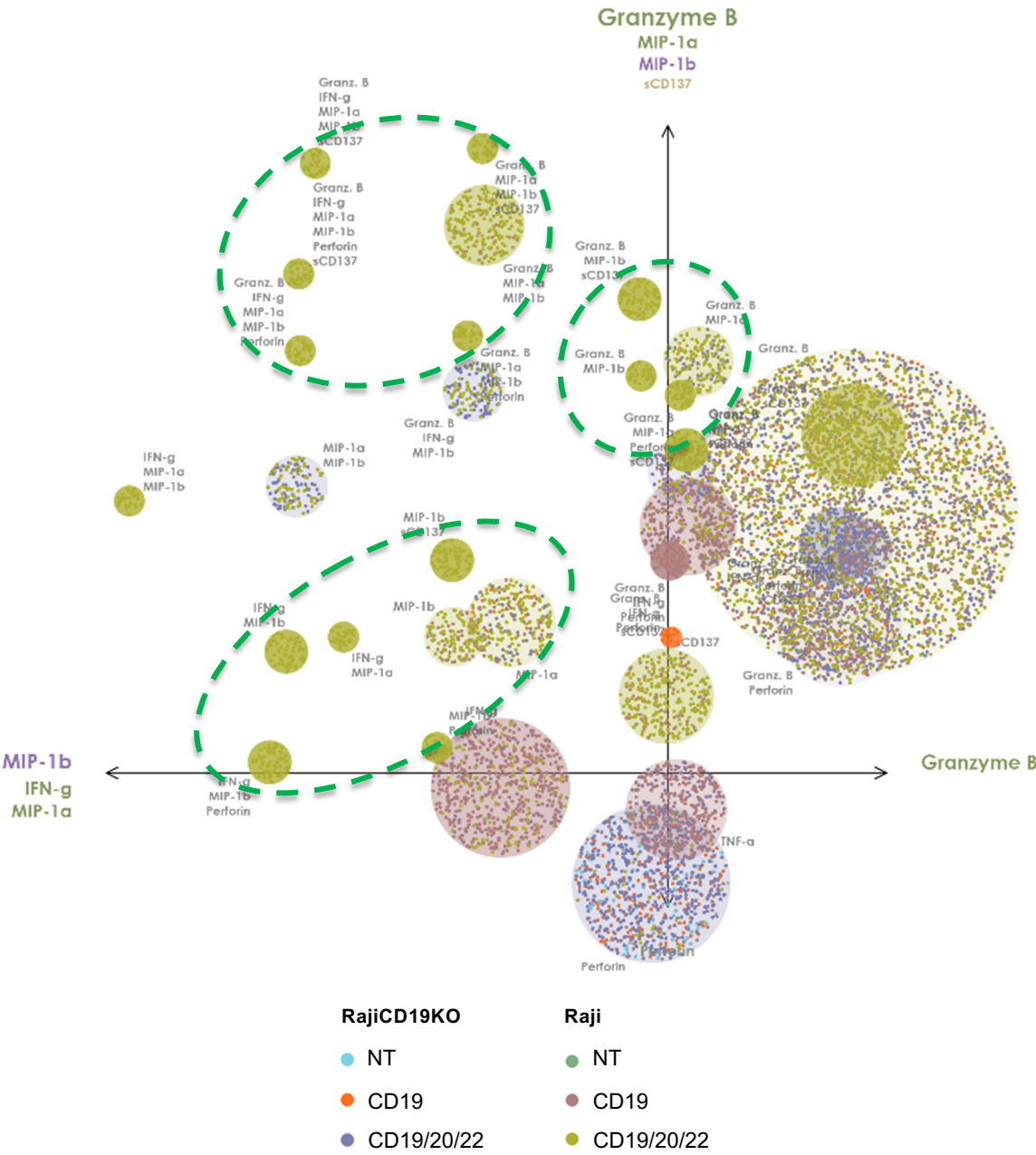

Supplement: Supplementary file 7 — Supplemental Figure 5 [file 41375_2020_792_MOESM7_ESM.pdf]

Supplemental Figure 6

A

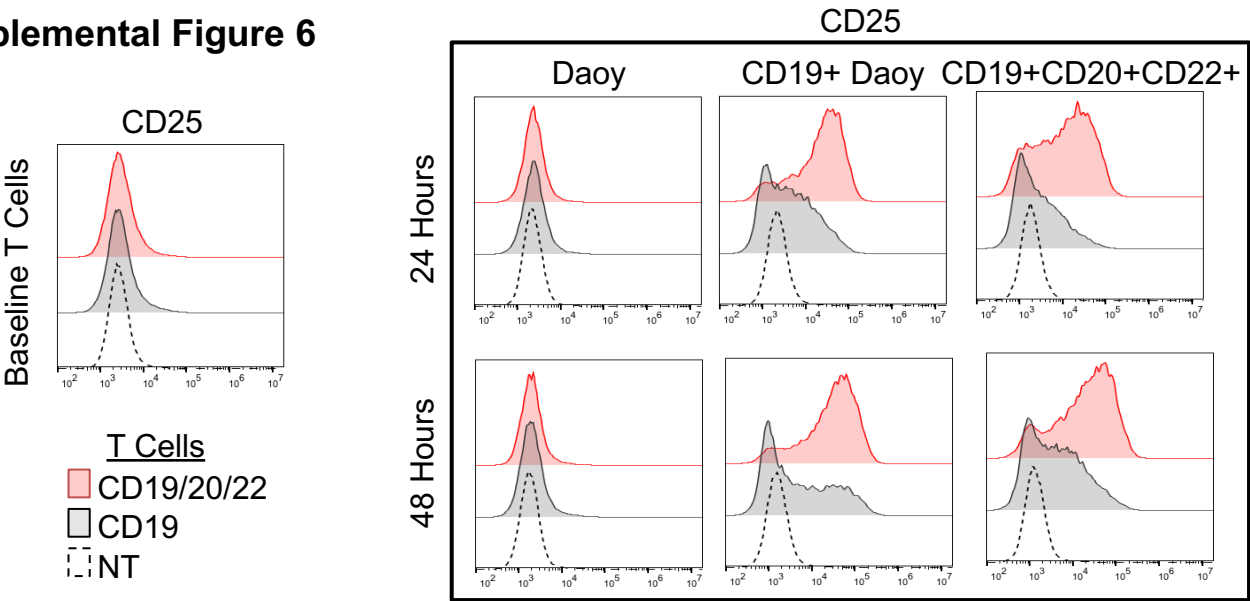

B

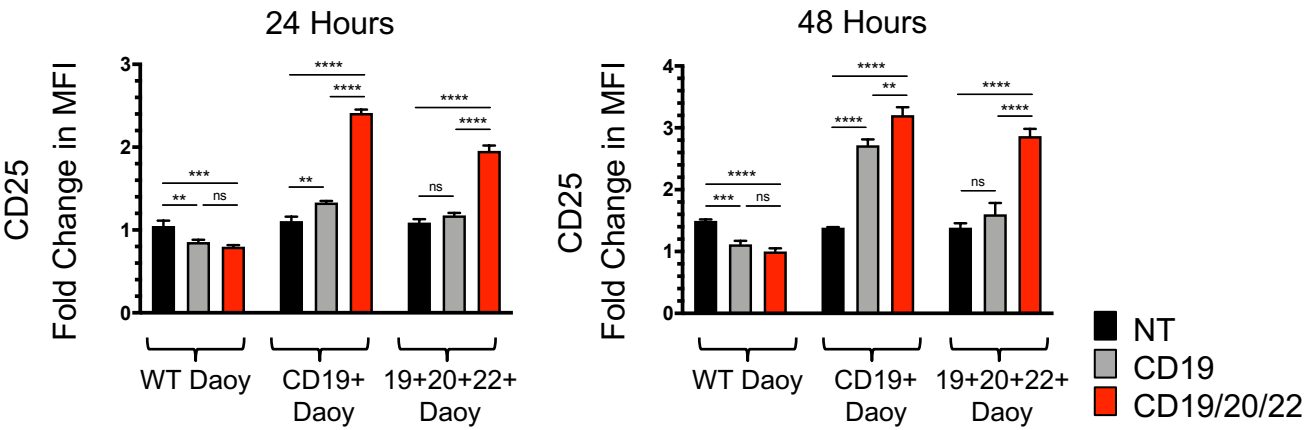

C

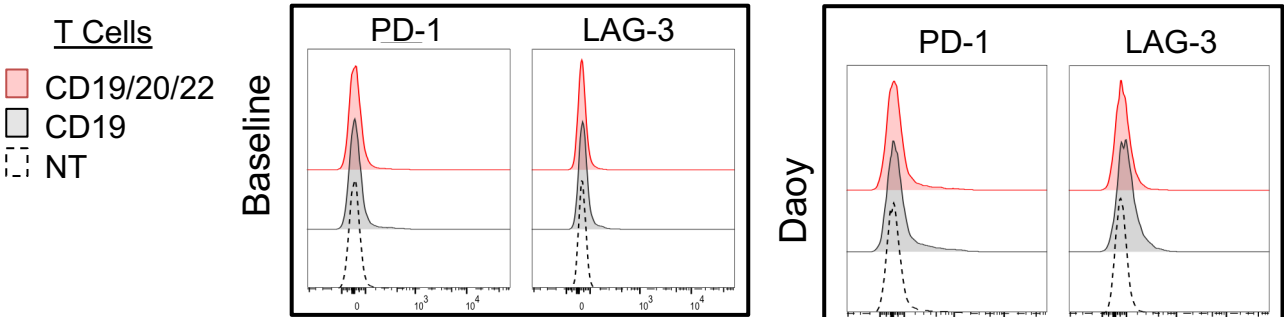

D

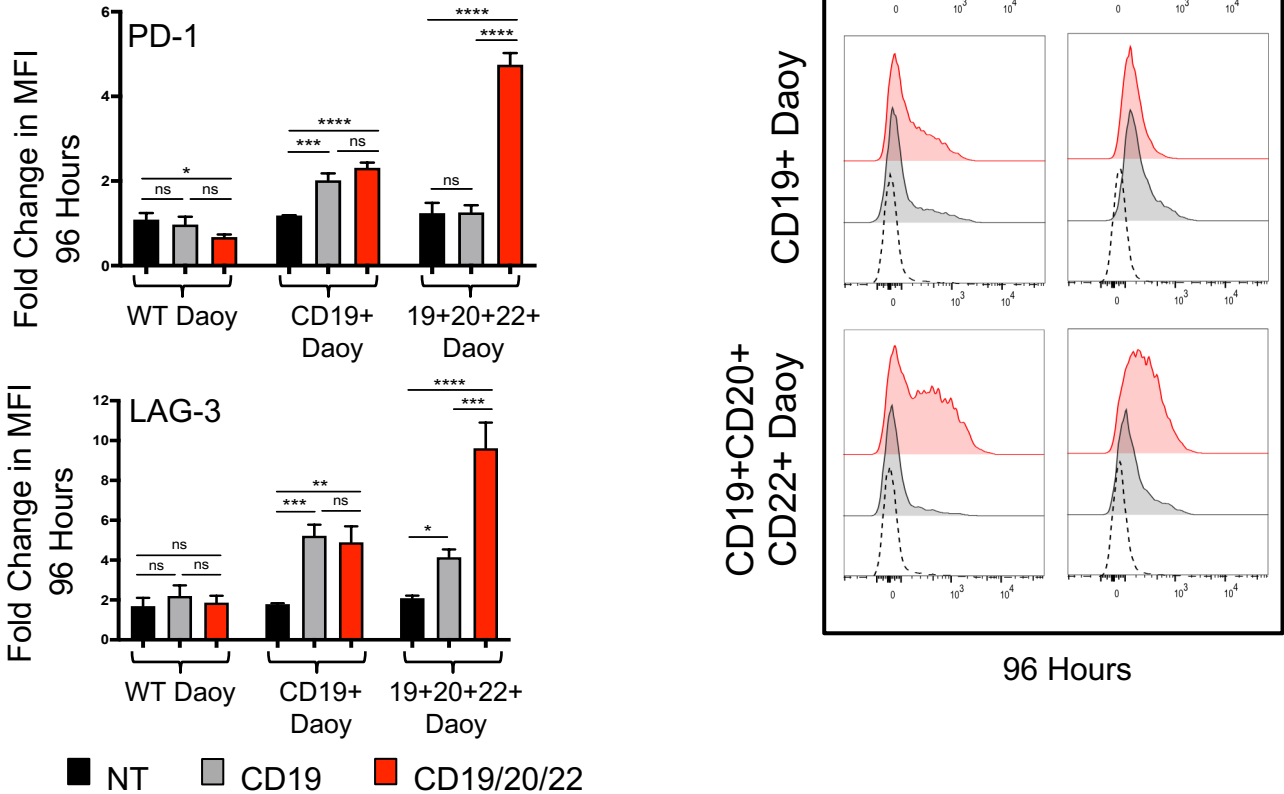

Supplement: Supplementary file 8 — Supplemental Figure 6 [file 41375_2020_792_MOESM8_ESM.pdf]
