# Supplementary material for: CAR T-cells that target acute B-lineage leukemia irrespective of CD19 expression
Source: Leukemia. 2020 Mar 24;35(1):75–89. doi: 10.1038/s41375-020-0792-2 (PMC7519582; doi:10.1038/s41375-020-0792-2)

Supplemental Figure 7

A

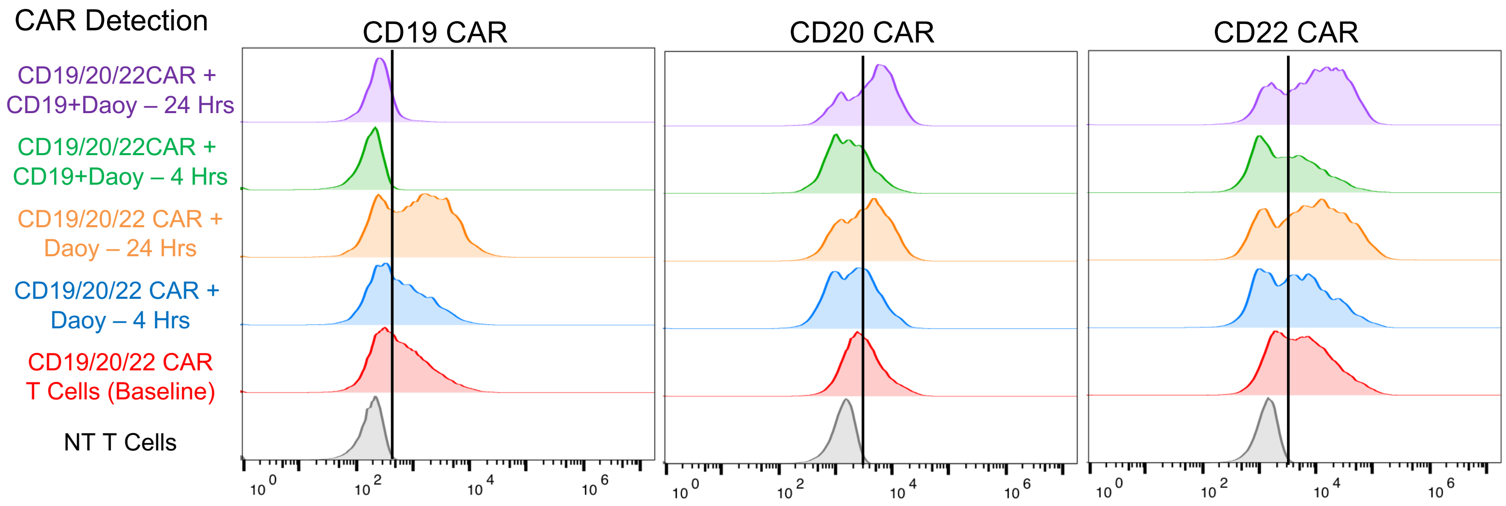

Supplement: Supplementary file 9 — Supplemental Figure 7 [file 41375_2020_792_MOESM9_ESM.pdf]
